# Assessment of the Microbiome and Potential Aflatoxin Associated With the Medicinal Herb *Platycladus orientalis*

**DOI:** 10.3389/fmicb.2020.582679

**Published:** 2020-10-23

**Authors:** Jingsheng Yu, Mengyue Guo, Wenjun Jiang, Meihua Yang, Xiaohui Pang

**Affiliations:** ^1^Key Lab of Chinese Medicine Resources Conservation, State Administration of Traditional Chinese Medicine of the People’s Republic of China, Institute of Medicinal Plant Development, Chinese Academy of Medical Sciences & Peking Union Medical College, Beijing, China; ^2^Engineering Research Center of Chinese Medicine Resource, Ministry of Education, Beijing, China

**Keywords:** Platycladi Semen, high-throughput sequencing, *Aspergillus*, aflatoxin, fungal microbiome, storage conditions

## Abstract

Platycladi Semen, which is derived from the dried ripe seed of *Platycladus orientalis*, has been used for the treatment of insomnia and constipation in China for 2000 years. However, it is susceptible to fungal and aflatoxin contamination under proper humidity and temperature during storage. Although aflatoxin contamination in Platycladi Semen has been reported preliminarily, few studies have been conducted on fungal infection and aflatoxin contamination simultaneously. Thus, this work aims to provide an in-depth understanding of fungal contamination in Platycladi Semen, and information on aflatoxin contamination. We focused on a comparison of the difference in fungal diversity between aflatoxin-contaminated and aflatoxin-free Platycladi Semen samples. First, aflatoxin levels in 11 Platycladi Semen samples, which were collected from local herbal markets in Shandong, Anhui, and Hebei provinces throughout China, were determined by IAC-HPLC-FLD, and positive confirmation of detected samples was performed by LC-MS/MS. The samples were divided into two groups, based on production or non-production of aflatoxin. We then used the Illumina MiSeq PE250 platform, and targeted the internal transcribed spacer two sequences to analyze the diversity and composition of the fungal microbiome, as well as to assess the presence of potential mycotoxin-producing fungi. Results showed that five samples were contaminated with aflatoxins, one of which exceeded the legal limits of Chinese Pharmacopeia Commission (2015). At the phylum level, the Ascomycota was the most dominant in all tested samples, with a relative abundance of 83.04–99.46%. *Aspergillus* (27.88–97.28%), *Xerochrysium* (0–28.49%), and *Xeromyces* (0–22.24%) were the three predominant genera. Furthermore, differences in fungal composition between the aflatoxin-contaminated and aflatoxin-free groups, as well as between different provinces were observed. A total of 74 species were identified, and four potential mycotoxin-producing fungi were detected in all samples, namely *Aspergillus flavus*, *Aspergillus fumigatus*, *Fusarium poae*, and *Penicillium steckii*. In conclusion, we report the great diversity of fungi associated with Platycladi Semen, highlight the risk to consumers of ingesting potent aflatoxin, and provide a reference for the safe application and quality improvement of Platycladi Semen.

## Introduction

The use of herbs is an integral part of Chinese culture. Due to their nutritional and medicinal applications they have played an important role in the development of global health industries. It has been reported that about 70–80% of world’s population has at least utilized herbal medicines in their primary healthcare (Chan, 2003). They have been used to treat major epidemic diseases in China, especially COVID-19. Some herbs, such as *Panax ginseng* and *Ginkgo biloba*, have become important dietary supplements in daily life (Mancuso and Santangelo, 2017; Tian et al., 2017). Platycladi Semen (PS, Bai Zi Ren in Chinese) is derived from the dried ripe seed of *Platycladus orientalis*, which has been used for the treatment of insomnia and constipation in China for over 2000 years (Chinese Pharmacopeia Commission, 2015). Modern pharmacological studies have shown its beneficial effects on the human nervous system as well as its antioxidant potential (Ju et al., 2010; Alamadri et al., 2018). However, fungal contamination in herbs is a common problem that affects their quality and safety. In the United States, Tournas et al. (2013) indicated that the total counts of fungi in tested milk thistle samples ranged between <2.00 and 5.60 log_10_ colony forming units per gram. Additionally, the potential for these fungi to produce toxic secondary metabolites (i.e., mycotoxins) as natural contaminants pose serious threats to consumer health. The occurrence of mycotoxins in herbs has been reported in many countries. A Pakistani survey on the contamination of aflatoxin (AF) and ochratoxin A in 30 herbal samples showed that the positive rates were 30 and 26.70%, respectively (Ahmad et al., 2014). Romagnoli et al. (2007) collected 103 samples of herbal infusions, aromatic herbs, and spices in Italy, and seven samples were contaminated with AF. Therefore, the severity of AF contamination in herbs cannot be underestimated. AF is one of the most serious mycotoxins reported in herbs worldwide, and is mainly produced by many species within the fungal genus *Aspergillus* (Williams et al., 2004). AF possesses carcinogenic properties that not only cause liver, stomach, and lung cancer, but also anemia, jaundice, and hepatotoxicity. AFB_1_ is the most toxic and carcinogenic natural compound known in AF group, and has been considered as the Group I carcinogen by the International Agency for Research on Cancer [IARC], 1993. PS is highly susceptible to contamination by AF. Therefore, the presence of the AF must be examined before PS is marketed (Chinese Pharmacopeia Commission, 2015). Chen et al. (2015) detected the AF level in five PS samples, and observed that the AFB_1_ level in PS was up to 52 μg/kg, exceeding the legal level of the Chinese Pharmacopeia Commission (2015). Furthermore, they indicated that the improper storage conditions (i.e., water-activity) might result in high fungal infection and AF contamination. Thus, the safety of PS during storage should be considered important.

As a result of the AF contamination in herbs and food, legal limits were set worldwide. The Chinese Pharmacopeia Commission has set respective limits of AFB_1_ and total AF at 5 and 10 μg/kg (Chinese Pharmacopeia Commission, 2015). The European Commission Regulation (1998) has set maximum limits of total AF and AFB_1_ at 4 and 2 μg/kg in cereals, respectively. Current detection methods for AF level include thin layer chromatography, enzyme-linked immune sorbent assay, and liquid chromatography-mass spectrometry (Wang et al., 2013; Zitomer et al., 2015; Akhund et al., 2017; Annunziata et al., 2017). The purification of AF by high-performance liquid chromatography-fluorescence detection combined with immunoaffinity column (IAC-HPLC-FLD) has been applied in many complex sample substrates, which is easy to operate and highly sensitive. Thus, AF contamination in PS samples can be detected through IAC-HPLC-FLD. Another method of AF detection with proven accuracy is liquid chromatography with tandem mass spectrometry. Thus, positive results of AFB were confirmed through LC- MS/MS.

Given the continuous development in genomic technologies, high-throughput sequencing is widely applied in various areas. It provides insights into the diversity of microorganisms from different sources (Fan et al., 2018; Ezekiel et al., 2019). Traditionally, fungal identification from herbs has involved macro- and micro-morphological examinations through culture work and microscopy. However, the application of traditional methods in identifying fungi is tedious and time consuming work, especially when attempting to assess fungal community structure. Additionally, many fungal strains cannot grow in synthetic medium, causing them to be overlooked. Moreover, misidentifications are possible due to the complex phenotypes of some fungi. Therefore, a precise and effective technology is needed. High-Throughput Sequencing (HTS) provides a new and useful strategy for the analysis of fungal mycobiomes present in herbs.

In this study, we conducted obligatory AF testing in 11 samples of the medicinal herb, *Platycladus orientalis* sampled in three Chinese provinces. We then tested the feasibility of HTS for the rapid detection of mycotoxigenic fungi in our samples. Finally, comparisons of fungal diversity were assessed between two groups (AF-contaminated versus AF-free), as well as between the three different provinces. To our knowledge, this is the first time to study the fungal microbiome in PS through HTS combined with AF detection. It should provide a reference for the design and implementation of fungal and AF management strategies for this important medicinal herb.

## Materials and Methods

### Sample Collection

Eleven PS samples were collected from local herbal markets in Shandong (*n* = 8), Anhui (*n* = 2), and Hebei (*n* = 1) provinces throughout China. During the collection process, we found far less production of PS in the other provinces. In China, PS is mainly produced in Shandong province. Thus, most of the samples were collected from Shandong province. In each market, PS was stored in open containers. We purchased 500 g per sample, and placed it into a sterile paper bag. All samples were transported to the Institute of Medicinal Plant Development, Chinese Academy of Medical Sciences, where Professor Meihua Yang confirmed their identities as PS, and they were assigned voucher numbers (BZ1-BZ11).

### Aflatoxin Assays of PS Samples

The mixed standard solution (AFB_1_ 2.00 μg/mL, AFB_2_ 0.50 μg/mL, AFG_1_ 2.00 μg/mL, and AFG_2_ 0.50 μg/mL) was purchased from Supelco (United States). A series of working standard solutions (0.25–25 μg/L for AFB_1_, 0.0625–6.25 μg/L for AFB_2_, 0.25–25 μg/L for AFG_1_, and 0.0625–6.25 μg/L for AFG_2_) were prepared with the solution of methanol–water (50:50, v/v). Five grams of finely pulverized sample was mixed with 1 g sodium chloride, and was extracted by ultrasonication with 25 mL of methanol: water (70:30, v/v) for 15 min, and then centrifuged for 5 min (at 10000 g). Five milliliters of supernatant was diluted by adding 45 mL of pure water, and filtered through a Whatman paper (0.45 μm). Following the cartridge instructions, 40 mL of sample extract was loaded on the HCM0125 IAC (Meizheng Biotechnology Co., Ltd., Beijing, China). The IAC was then rinsed with 10 mL water (3 mL/min). After, the column was washed with methanol (0.5–1 mL/min), and the AF was eluted with 2 mL of methanol in a dark flask. The eluate was dried under a stream of N_2_ to evaporate the methanol at 50°C, and the residue was re-dissolved in 1 mL of methanol–water (50: 50, v/v). The solution was vortexed for 30 s, and filtered through a 0.22 μm glass microfiber filter for chromatographic detection.

Chromatographic analysis to detect AF was performed on a Shimadzu LC-20 AT HPLC system (Shimadzu, Kyoto, Japan) consisting of a RF-10A_XL_ fluorescence detector. The separation was performed on a Cloversil ODS-U column (4.6 mm × 250 mm, 5 μm), and the column temperature was kept at 30°C. The mobile phase consisted of methanol-acetonitrile-water (40:18:42, v/v/v) at a flow rate of 1 mL/min, and the injection volume was 10 μL. The eluate was monitored by a fluorescence detector. Fluorescence conditions were optimized for AF (excitation 360 nm and emission 440 nm wavelengths). To confirm and quantify AF in our samples, a Shimadzu LC system coupled with a QTRAP^®^ 5500 mass spectrometer (Applied Biosystems, Foster City, CA, United States) was used for LC-MS/MS analysis. The separation was performed on a CAPCELL CORE C_18_ column (100 mm × 2.10 mm, 2.70 μm). The mobile phase consisted of A: methanol (containing 0.1% formic acid) and B: 50 mM ammonium acetate solution (containing 0.1% formic acid) at a flow rate of 0.30 mL/min. The gradient elution procedure was as follows: 0.01–0.50 min 60% B, 0.50–4.50 min (60–5%, B), 4.50–6.50 min 5% B, 6.50–6.51 min (5–60%, B), and 6.51–10.00 min 60% B. The injection volume was 2 μL. The mass spectrometer was operated in the positive ESI mode with multiple reaction monitoring. Nitrogen was used as the nebulizer, heater, curtain gas, and collision activation dissociation gas. For AFB_1_ and AFB_2_, ion transitions were simultaneously monitored at *m/z* 313→285, *m/z* 313→254 and *m/z* 315→287, *m/z* 315→259. Optimal parameters were as follows: GS1, GS2, and curtain gases were 55, 55, and 35 psi, respectively; ion spray voltage was 4500 V; ion source temperature was set at 500°C. The declustering potential was 110 V for AFB_1_ and 170 V for AFB_2_. For AFB_1_, collision energy was 32 eV for *m/z* 313→285 and 43 eV for *m/z* 313→254. For AFB_2_, collision energy was 36 eV for *m/z* 315→287 and 40 eV for *m/z* 315→259. Samples would be divided into two groups according to whether or not AF was detected, namely BC (non-aflatoxigenic) or CD (aflatoxigenic).

### Analysis of Fungal Microbiome Diversity in 11 PS Samples

DNA extraction and PCR protocols for this study closely followed a previous report by Guo et al. (2018) with some modifications. Briefly, 2.50 g of each PS sample was transferred into a 15 mL sterilized centrifuge tube. We isolated total DNA from microorganisms following the instructions bundled with the EZNA^®^ soil DNA kit (Omega Bio-tek., Inc., Norcross, GA, United States). The DNA was stored at −20°C until it was time to proceed to the PCR step. We acquired primers of ITS3 (5′-GCATCGATGAAGAACGCAGC-3′) and ITS4 (5′-TCCTCCGCTTATTGATATGC-3′) to amplify the internal transcribed spacer 2 (ITS2) region (White et al., 1990). The PCR products were analyzed on a 2% agarose gel, purified and qualified for the desired fragment. Purified ITS2 amplicons were sequenced using the Illumina MiSeq PE250 platform (Illumina, United States) by AuwiGene Technology Co., Ltd. (Beijing, China). The raw sequences were uploaded to the National Center for Biotechnology Information Sequence Read Archive database.

Raw FASTQ files were de-multiplexed and quality-filtered by Trimmomatic (v 0.36) and Pear (v 0.9.6) software. The reads were truncated at any site, receiving an average quality score of <20 over a 50 bp sliding window. We merged in the sequences that overlapped by more than 10 bp. The sequences were clustered into operational taxonomic units (OTUs, 97% similarity) by using UPARSE (version 7.1^1^), and chimeras were removed by using USEARCH (v8.1.1861)^2^ (Edgar, 2010, 2013). Each OTU was annotated in accordance with the UNITE database at different levels, ranging from species to kingdom. In order to ensure the accuracy of the OTU annotation, we verified taxonomical classification of all OTUs via manual BLAST search in the International Nucleotide Sequence Database Collaboration. A rarefaction curve was constructed to reflect normalization to even depths across samples in QIIME. A flattened curve indicated that the sequencing data volume was reasonable.

For our fungal microbiome comparisons, we grouped samples in two different ways: one was based on the presence/absence of aflatoxin, and the other was based on sampling province. The α-diversity was estimated through three indices, namely Chao1, Good’s coverage, and Shannon. The weighted and unweighted UniFrac distance matrices were used to estimate β-diversity. The groups of samples were subjected to Principal co-ordinates analysis (PCoA) and partial least squares discrimination analysis (PLS-DA) to explore their dissimilarity (Edgar, 2010). Moreover, the samples were hierarchically clustered based on β-diversity matrices using the unweighted pair group method with arithmetic mean (UPGMA). Venn analysis was conducted, and heatmaps were inferred to perform distance calculation and cluster analyses using R tools (Jami et al., 2013).

## Results

### Aflatoxin Assays of 11 PS Samples

We detected aflatoxin in five of the 11 PS samples (BZ2, BZ3, BZ8, BZ9, and BZ10) having an over-standard rate of 9.10% (Table 1). Their AFB_1_ levels ranged from 1.22 μg/kg (BZ3) to 23.17 μg/kg (BZ2). BZ2 was the only sample that far exceeded the legal limits set by the Chinese Pharmacopeia Commission (2015). Measurable AFB_2_ was only detected in BZ2 (1.84 μg/kg) although three other samples showed trace amounts that were less than the limit of quantification (LOQ). BZ8 had measurable levels of AFG_1_ (2.09 μg/kg), while BZ2 had only trace amounts of this mycotoxin below the LOQ. No other samples revealed the presence of AFG_1_. BZ8 also had trace amounts of AFG_2_, which were lower than the LOQ. Total AF levels ranged from 1.22 μg/kg (BZ3) to 25.01 μg/kg (BZ2). The linear regression equation, correlation coefficient, range of linearity, LOD, and LOQ are shown in Table 2, and HPLC and LC-MS/MS chromatograms for our samples are shown in Supplementary Figures S1,S2, respectively.

**TABLE 1:** Occurrence and levels of AF in tested PS samples.

Voucher No.	Sampling Location	AFG_2_ (μg/kg)	AFG_1_ (μg/kg)	AFB_2_ (μg/kg)	AFB_1_ (μg/kg)	AFs (μg/kg)
BZ1	Anhui province	ND	ND	ND	ND	ND
BZ2	Hebei province	ND	<LOQ	1.84	23.17	25.01
BZ3	Shandong province	ND	ND	ND	1.22	1.22
BZ4	Shandong province	ND	ND	ND	ND	ND
BZ5	Shandong province	ND	ND	ND	ND	ND
BZ6	Shandong province	ND	ND	ND	ND	ND
BZ7	Shandong province	ND	ND	ND	ND	ND
BZ8	Shandong province	<LOQ	2.09	<LOQ	1.57	3.66
BZ9	Shandong province	ND	ND	<LOQ	3.94	3.94
BZ10	Shandong province	ND	ND	<LOQ	2.10	2.10
BZ11	Anhui province	ND	ND	ND	ND	ND

*ND indicates no aflatoxin was detected. LOQ indicates trace aflatoxin was detected below the limit of quantitation.*

**TABLE 2:** Linearity and sensitivity data for aflatoxin standards used for this study.

Analytes	Linear equation (*y* = *m*x + *n*)	r	Range (μg/L)	LOD (μg/kg)	LOQ (μg/kg)
AFB_1_	*y* = 36325x + 192.24	0.9996	0.25–25	0.40	1.22
AFB_2_	*y* = 81614x + 759.41	0.9996	0.0625–6.25	0.10	0.33
AFG_1_	*y* = 23159x + 1424.6	0.9996	0.25–25	0.56	1.50
AFG_2_	*y* = 50215x + 1435.1	0.9996	0.0625–6.25	0.11	0.33

**y*, peak area; x, concentration; *m*, slope; *n*, intercept.*

### Composition and Diversity of Fungal Microbiomes in 11 PS Samples

**FIGURE 1:**
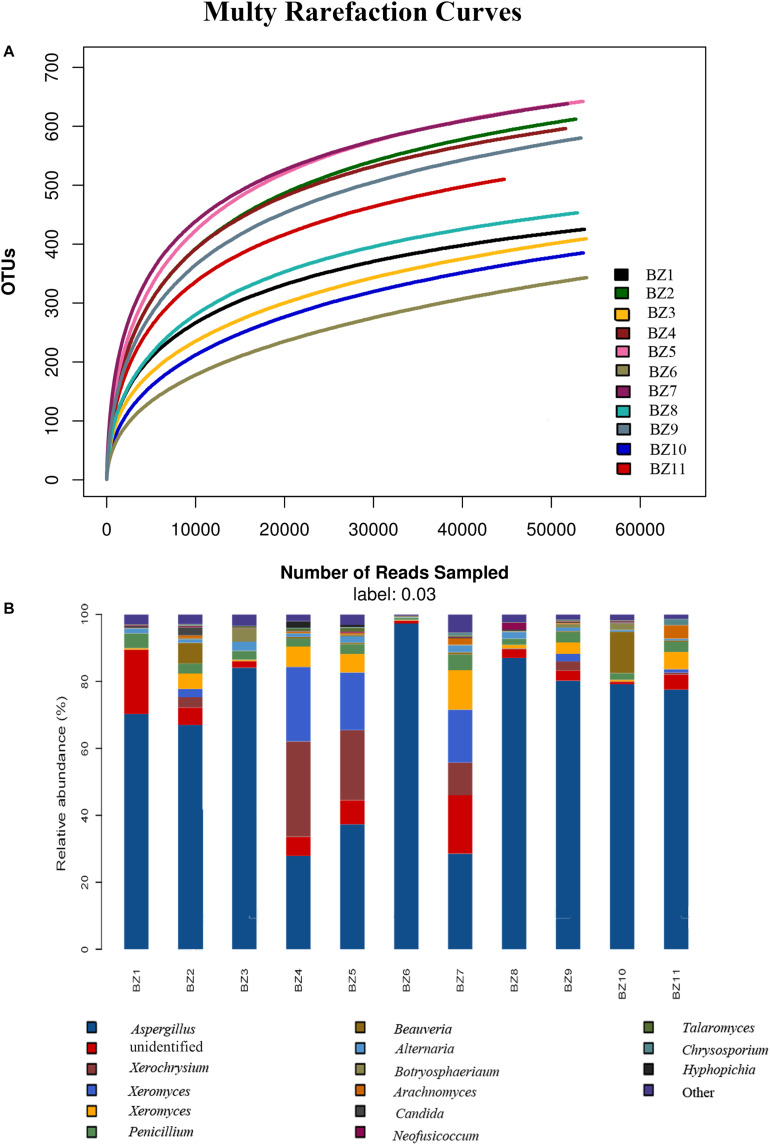
Microbiome composition analyses for the fungal genera present in 11 PS samples: **(A)** Rarefaction curves for OTUs in each PS sample to even sampling depth; **(B)** Composition at the genus level for each PS sample.

A total of 897,917 ITS2 sequences (200–500 bp) were obtained after excluding the chimeric sequences. Taxonomic assignment of the sequences with ≥97% similarity in the UNITE database resulted in 881 OTUs. The raw sequences uploaded to the National Center for Biotechnology Information Sequence Read Archive database were assigned accession numbers SAMN13722171- SAMN13722181 (Table 3). Supplementary Table S1 lists the distributions of OTUs across our 11 PS samples. Rarefaction curve analysis resulted in our 11 PS samples as homogeneously parallel to the x-axis, indicating the reliability of the sequencing depth employed. The highest and lowest numbers of OTUs were detected in BZ5 and BZ6 (Figure 1A). All 881 OTUs were subjected to BLAST confirmation. In all PS samples (Supplementary Table S1), Ascomycota was the dominant phylum, accounting for 83.04–99.46% of the OTUs. Two other phyla, Basidiomycota and Mucoromycota, as well as OTUs unidentifiable at this level, were detected at low relative abundance. Further taxonomical classification at the class level showed that Eurotiomycetes (63.47–98.04%) predominated among the other 17 classes identified in all PS samples. Thirty-three OTUs were unidentifiable at class level. At the order level, the relative abundance of Eurotiales (60.72–98.03%) was greatest among 46 identifiable orders, with 51 unidentifiable OTUs. A total of 207 OTUs were subjected to BLAST confirmation at the genus level. Moreover, 62 OTUs could be identified at the genus level via manual BLAST search, dominated by *Aspergillus* having a relative abundance of 27.88–97.28% (Figure 1B). There were 139 OTUs unidentifiable at the genus level. The top 15 abundant genera are shown in Figure 1B. Although the sequences were clustered into 881 OTUs, many OTUs could only be identified at genus or higher level. There were 220 OTUs that could not be resolved to species level. The UNITE identified 107 fungal taxa at the species level. However, 74 fungal taxa could be identified at the species level via manual BLAST search. Four potential mycotoxin-producing fungi were detected in all PS samples, namely *Aspergillus flavus*, *Aspergillus fumigatus*, *Fusarium poae*, and *Penicillium steckii*. With regard to aflatoxigenic species, five were represented in at least one sample. Three that produce only B aflatoxins (*Aspergillus flavus*, *Aspergillus bombycis*, and *Aspergillus pseudotamarii*) and two that produce B + G aflatoxins (*Aspergillus parasiticus* and *Aspergillus nomius*). Only *Aspergillus flavus* was represented in more than one OTU.

**TABLE 3:** Sampling locations, aflatoxin profiles, and GenBank accession numbers for 11 PS samples.

Voucher No.	Sampling Location	AF Profile^a^	GenBank Accession No.
BZ1	Anhui province	BC	SAMN13722171
BZ2	Hebei province	CD	SAMN13722172
BZ3	Shandong province	CD	SAMN13722173
BZ4	Shandong province	BC	SAMN13722174
BZ5	Shandong province	BC	SAMN13722175
BZ6	Shandong province	BC	SAMN13722176
BZ7	Shandong province	BC	SAMN13722177
BZ8	Shandong province	CD	SAMN13722178
BZ9	Shandong province	CD	SAMN13722179
BZ10	Shandong province	CD	SAMN13722180
BZ11	Anhui province	BC	SAMN13722181

*^*a*^Aflatoxin profile for each sample is either non-aflatoxigenic (BC) or aflatoxigenic (CD).*

### Comparison of Fungal Microbiomes Based on the Presence or Absence of Aflatoxin

**FIGURE 2:**
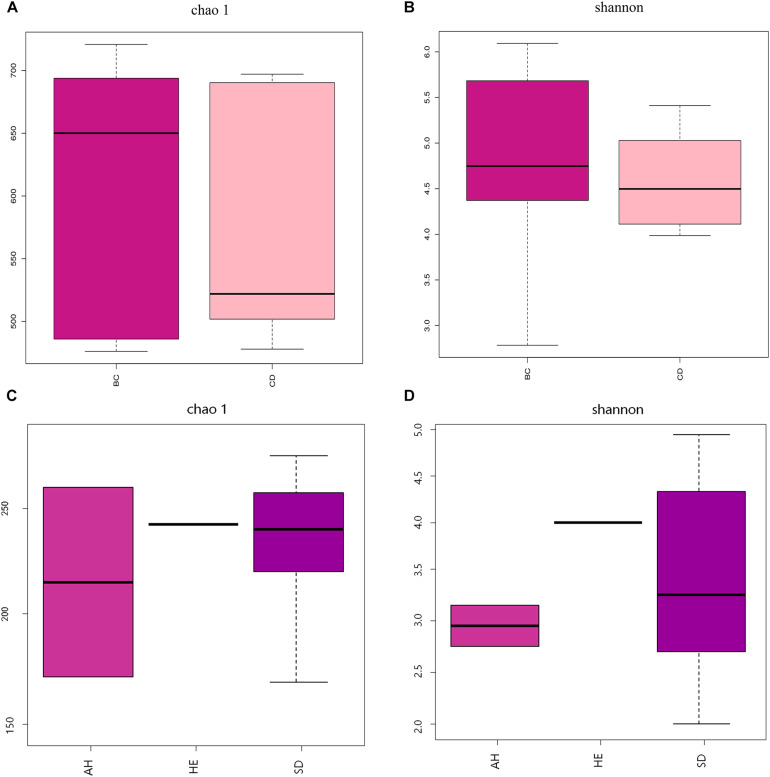
Analysis of fungal diversity across 11 PS samples, using Chao 1 **(A,C)** and Shannon **(B,D)** α-diversity indices, based on two groupings: absence (BC) or presence (CD) of aflatoxin, and sampling province (AH, HE, or SD). AH = Anhui, HE = Hebei, SD = Shandong.

**FIGURE 3:**
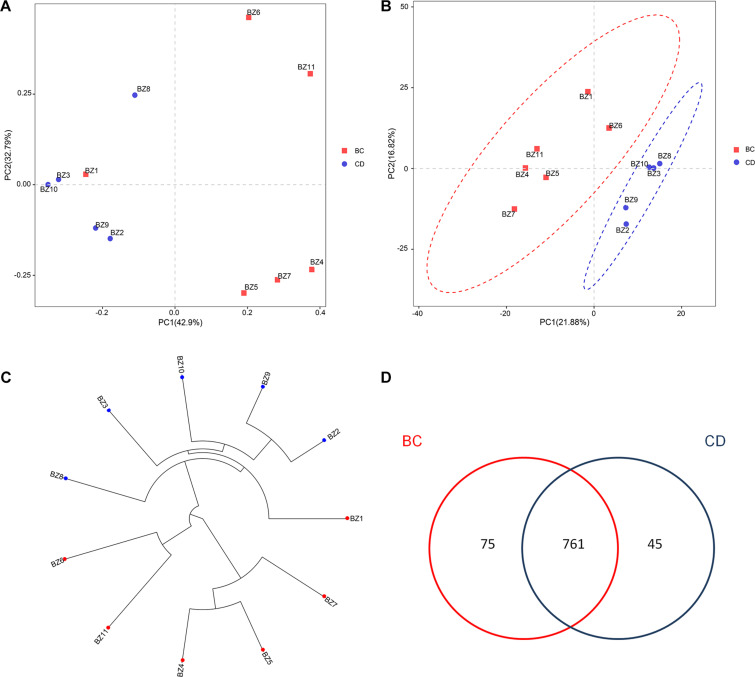
Analysis of the β-diversity for fungal microbiomes across 11 PS samples based on grouping by absence (BC) or presence (CD) of aflatoxin: **(A)** PCoA plot based on weighted UniFrac distance matrices; **(B)** PLS-DA plot based on weighted UniFrac distance matrices; **(C)** UPGMA clustering based on unweighted UniFrac distance analysis; **(D)** Venn diagram of shared and unique OTUs.

**FIGURE 4:**
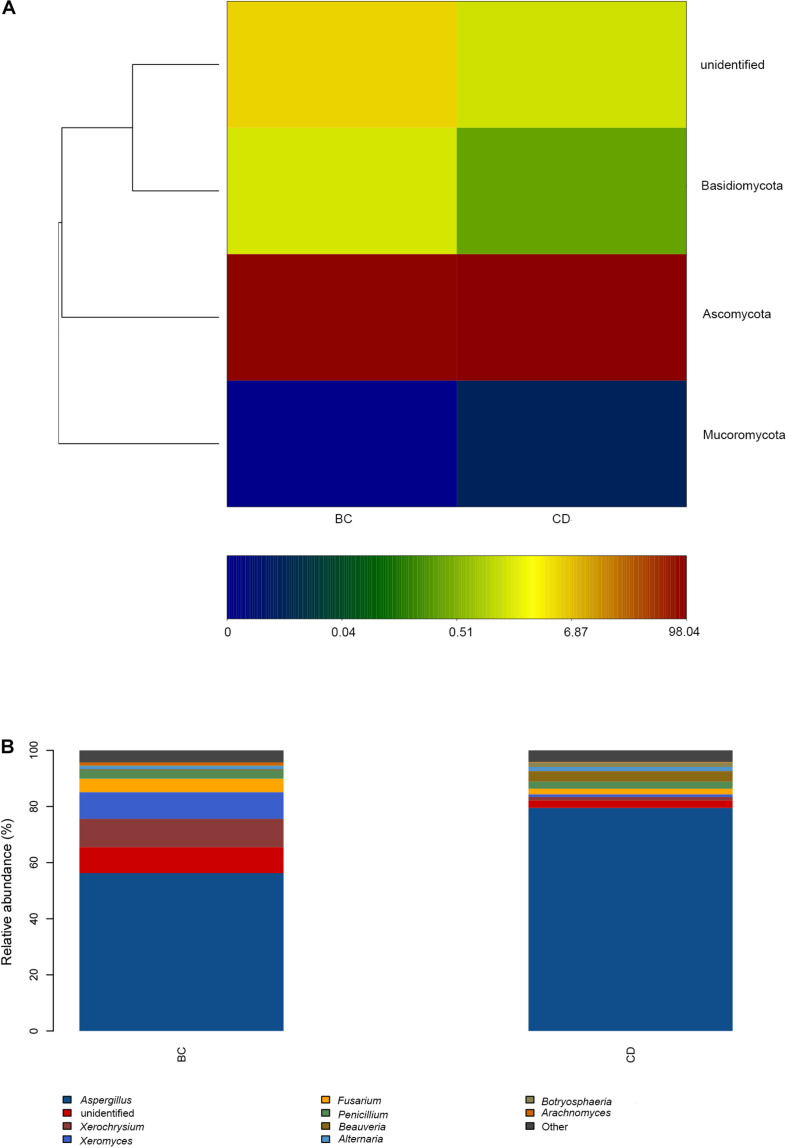
Microbiome composition analyses for observed fungal phyla and genera in our PS samples based on two groupings by absence (BC) or presence (CD) of aflatoxin: **(A)** Composition at the phylum level; **(B)** Composition at the genus level.

After determining that five samples contained AF, we compared fungal microbiome diversity across the AF-free (BC) samples (836 OTUs) to that of the AF-contaminated (CD) samples (806 OTUs). Figures 2A,B showed that both Shannon and Chao1 indices for the BC group were higher (i.e., greater diversity) than those for the CD group. In addition, Good’s coverage index yielded estimates of over 99.70% in all samples (Supplementary Table S2), indicating good overall sampling. For β-diversity, PCoA and PLS-DA (Figures 3A,B, respectively) indicated that BC and CD groups showed different degrees of clustering. Except for BZ1, which was grouped with the CD group in the PCoA plot, the result of hierarchical clustering analysis was consistent with β-diversity analysis, and two groups were significantly distinguishable with exception of BZ1, which again grouped with the CD samples (Figure 3C, *R* = 0.3653, *p* = 0.023). Venn analysis revealed 761 shared OTUs between the two groups, with BC and CD each having 75 and 45 unique OTUs, respectively (Figure 3D). Heatmaps (Figure 4) for the relative abundances fungal microbiomes in the BC and CD groups support the findings of our diversity indices. Figures 4A,B show pooled community composition differences (at phylum and genus level, respectively) between the BC and CD groups, and overall greater phylum diversity was observed in the BC group (for the unidentified phyla, Basidiomycota and Mucoromycota). The relative abundance of Ascomycota seemed equal in both groups. Based on the cluster analysis of OTUs in BC and CD groups, the relationship of the unidentified phyla and Basidiomycota was closer than that of Ascomycota and Mucoromycota. The presence of Ascomycota was lower (92.84%) in the BC group than in the CD group (98.04%). The relative abundance of Basidiomycota was higher in the BC group (1.73%) than in the CD group (0.38%). By contrast, the relative abundance of Mucoromycota was lower in the BC group (0.00%) than in the CD group (0.01%). With regard to fungal genera, the relative abundance of *Aspergillus* was lower in the BC group (56.26%) than that in the CD group (79.51%), as were the numbers of *Beauveria*. By contrast, the relative abundances of *Xerochrysium* and *Fusarium* were higher in the BC group (10.10 and 4.79%, respectively) than in the CD group (1.17 and 2%, respectively), as were the unidentified genera and *Penicillium*. At the species level, the proportion of potential mycotoxin-producing fungi (not restricted to aflatoxigenic fungi) was higher in the CD group (31.89%) than the 10.95% observed in the BC group.

### Comparison of Fungal Microbiomes Based on Sampling Location

**FIGURE 5:**
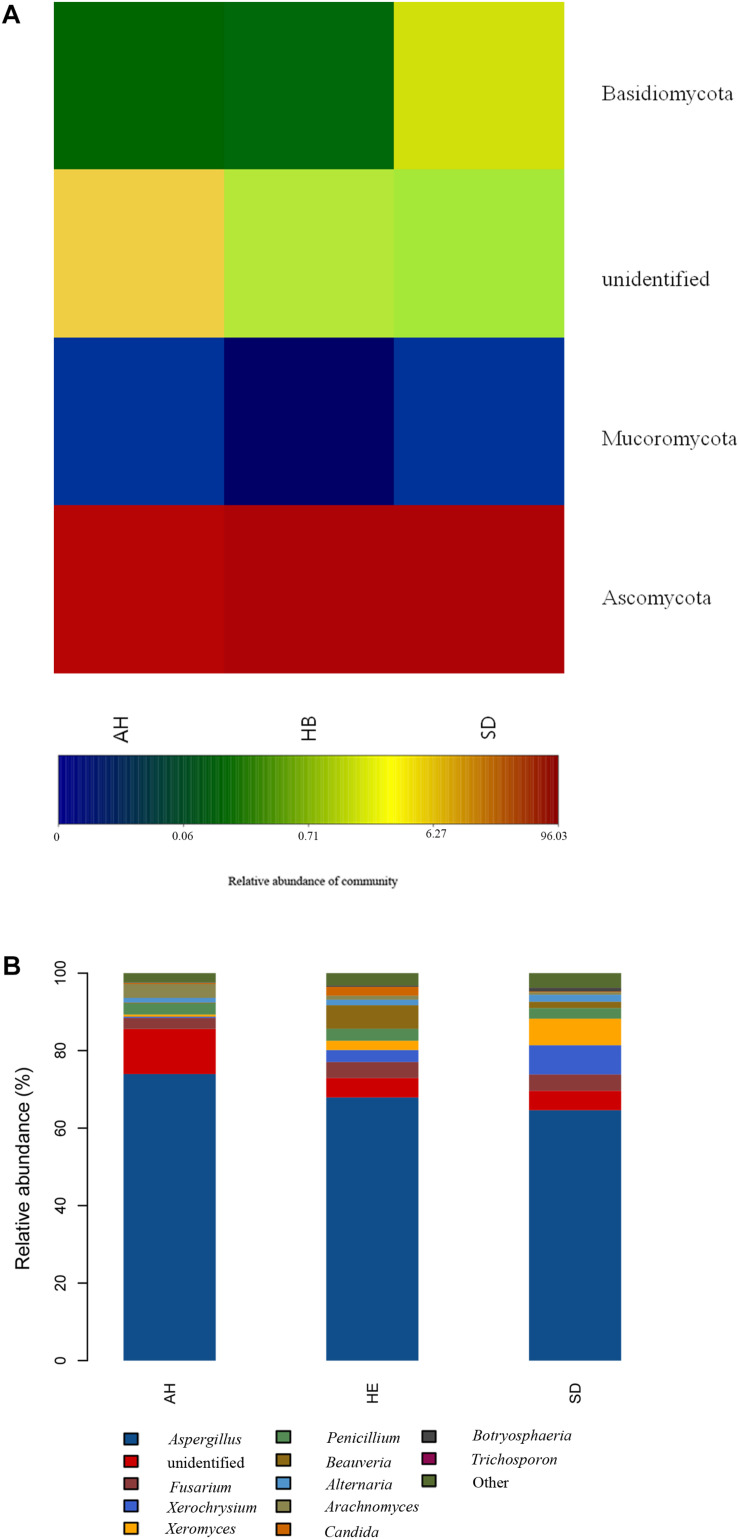
Microbiome composition analyses for the fungal phyla and genera present in each sampling province: **(A)** Composition at the phylum level; **(B)** Composition at the genus level.

Pooling OTUs based on sampling location, there were 857 from Shandong province, 730 from Anhui province, and 612 from Hebei province. Figures 2C,D showed that both Shannon and Chao1 indices for the samples from Hebei province were higher (i.e., greater diversity) than those for the samples from Anhui and Shandong provinces. Figures 5A,B show pooled community composition differences (at phylum and genus level, respectively) between different provinces. Compared with those in the samples from Hebei and Shandong provinces, greater unidentified phyla diversity was observed in the samples from Anhui province, as well as lower Ascomycota diversity. At the phylum level, Ascomycota predominated with PS samples from Shandong and Hebei having relative abundances of 96.43 and 96.36%, respectively. The relative abundance of Ascomycota in Anhui province was 89.98%. The relative abundances of Basidiomycota in Shandong province, Ahui province, and Hebei province were 1.41, 0.31, and 0.27%. The presence of Mucoromycota was also detected in three provinces at low relative abundance. At the genus level, *Aspergillus* dominated in each province, and the average relative abundance of *Aspergillus* in Anhui province (73.90%) was higher than those in PS samples from Shandong province (65.18%) and Hebei province (66.99%, Table 4). The abundance of secondary and tertiary genera for each province exhibited more variability: *Xerochrysium* and *Xeromyces* in Shandong province; *Arachnomyces* and *Penicillium* in Anhui province; *Beauveria* and *Fusarium* in Hebei province. At the species level, the average relative abundances of the potential mycotoxin-producing fungi (*Aspergillus flavus*, *Aspergillus fumigatus*, *Fusarium poae*, and *Penicillium steckii*) detected in the samples from Anhui, Shandong, and Hebei provinces were 11.61, 31.04, and 21.08%.

**TABLE 4:** Relative abundance of ten most prominent fungal genera detected in different provinces.

Genus	Anhui province	Shandong province	Hebei province
*Aspergillus*	73.90%	65.18%	66.99%
*Xerochrysium*	0.30%	7.75%	3.07%
*Fusarium*	2.79%	3.63%	4.60%
*Xeromyces*	0.53%	7.21%	2.45%
*Penicillium*	3.67%	2.53%	3.01%
*Beauveria*	0.24%	1.79%	6.19%
*Alternaria*	0.99%	1.43%	1.05%
*Arachnomyces*	3.78%	0.40%	0.80%
*Candida*	0.30%	0.17%	2.46%
*Botryosphaeria*	0.09%	1.05%	0.32%

## Discussion

Mycotoxin contamination is a crucial and long-term concern in herbs. Main mycotoxins in herbs include AF, ochratoxins A, fumonisins, and T-2 toxin, among which AF is one of the most common. AF has received considerable concern from the public. With the development of processing technology and pharmaceutical industry, it is increasingly important to study the relationship between potential mycotoxin-producing fungi and storage conditions. In this study, *Aspergillus flavus* was dominant at the species level. The relative abundances of *Aspergillus flavus* in different samples were variable. Although the relative abundance of *Aspergillus flavus* was highest in BZ9 among all samples, the AFB level was not the highest. The reason may be that some *Aspergillus flavus* strains lack the aflatoxin biosynthesis cluster, causing them to be non-aflatoxigenic (Amaike and Keller, 2011). At the same time, storage conditions may not be conducive for AFB production. Many factors, including temperature, humidity, substrate, pH, nutrient availability, and packaging, may affect the growth of potential mycotoxin-producing fungi and the production of AF (Darko et al., 2018; Zhang et al., 2019). Among these factors, temperature and humidity are the most important parameters for fungal growth and AF production. A Chinese survey showed that favorable temperature and water activity for the growth of *Aspergillus flavus* were not always conducive for AFB_1_ production (Lv et al., 2019). Thus, the relationship between the storage condition and potential aflatoxin producing fungi should be studied further.

Fungal contamination associated with herbs has been reported worldwide (Romagnoli et al., 2007; Ahmad et al., 2014; Fabio and Armando, 2018). Due to the lack of standard management, fungal contamination in herbs may occur at any point in the production cycle, including cultivation, harvest, transport, and storage. The use of next-generation sequencing, in particular HTS, has attracted increasing interest to help reveal fungal contamination in herbs (Guo et al., 2018; Luo et al., 2019). In this study, a total of 3 phyla, 62 genera, and 74 species were identified through HTS. Ascomycota, Eurotiomycetes, and Eurotiales were the dominant phylum, class, and order. This was consistent with findings in other studies of fungal contamination in seed herbs (Guo et al., 2019; Jiang et al., 2020). *Aspergillus*, *Fusarium*, and *Penicillium* were the most common toxic genera in our PS samples, consistent with the results in previous studies (Kong et al., 2014; Guo et al., 2018, 2019; Luo et al., 2019; Jiang et al., 2020). *Aspergillus* spp. are common storage fungi, hence their prevalence in all of our samples (Krijgsheld et al., 2013). In addition to these genera, our study indicated that *Xerochrysium* and *Xeromyces* were also predominant in PS samples. Previous studies have reported that *Xerochrysium* and *Xeromyces* could catalyze substrates that release metabolic water to favor the growth of species in *Aspergillus* at low water-availability (Xing et al., 2016; Stevenson et al., 2017a,b). In addition, we analyzed the comparison of fungal microbiomes based on the presence or absence of aflatoxin. The richness and diversity of fungal microbiome in BC group were higher than those in CD group. It was consistent with the findings in other studies, and suggests that fungal microbiome diversity is reduced in the presence of AF contamination (Ding et al., 2015; Yuan et al., 2018). At the phylum level, the relative abundance of unidentified phyla in BC group was higher than that in CD group. Furthermore, the phylogeny result showed that the relationship between the unidentified phyla and Basidiomycota was close. The unidentified phyla may include Mortierellomycota, which has been reported to exist in seed or fructus herbs such as Cassiae Semen and *Lycium Ruthenicum* (Gao et al., 2019; Guo et al., 2019). Except for the BZ6 sample, the relative abundance of *Aspergillus* in CD group was higher than that in the BC group. The relative abundance of *Aspergillus* in BZ6 sample was 97.28%, which was the highest in all samples. Contrary to other samples, BZ6 was moldy during collection, which might be the reason for its high relative abundance of *Aspergillus*. However, the relative abundance of *Aspergillus flavus* (2.99%) in BZ6 was lower than the average relative abundances of *Aspergillus flavus* (14.39%) in CD group. Thus, although the relative abundance of *Aspergillus* was high, no AF level was detected in BZ6. In our initial assessment of AF presence, BZ1 was AF-free, and therefore it was assigned to group BC. However, in our PCoA and hierarchical clustering analyses, sample BZ1 was associating more closely with the AF contaminated samples (group CD). We also observed its fungal microbiome composition to be more similar to those of group CD samples, including the relative abundances of *Aspergillus* (70.25%), *Xerochrysium* (0.00%), and *Xeromyces* (0.00%). If we based our sample grouping on similarity of sample microbiomes, BZ1 could be re-assigned to the CD group despite being AF-free. Furthermore, its biosynthesis might be inhibited by other fungi including *Aspergillus niger* and *Alternaria alternate*, which were detected in BZ1. Both of these fungi have been reported that had inhibitory effect on AF biosynthesis of *Aspergillus flavus* (Xing et al., 2017; Gong et al., 2019). In addition, as a result of the highest AFB level, we analyzed the fungal composition of BZ2. The result showed that the relative abundance of *Aspergillus flavus* was lower than those in some samples of CD group (BZ9 and BZ10). It suggested that the aflatoxin production ability of *Aspergillus flavus* in BZ2 was stronger than those in BZ9 and BZ10. As for AFG contamination in BZ2 and BZ8, the result showed that both *Aspergillus parasiticus* and *Aspergillus nomius* were detected in BZ8, and only *Aspergillus nomius* was detected in BZ2. With regard to comparisons made per sampling region, although the highest concentration of *Aspergillus* was in PS samples from Anhui, no AF contamination was detected. The reason might be its low relative abundance of *Aspergillus flavus* (6.86%). By contrast, the relative abundance of *Aspergillus* in PS samples from Shandong province was the lowest among three provinces, and four out of seven samples from Shandong province (BZ3, BZ8, BZ9, and BZ10) were contaminated with AF. Perhaps the higher number of samples diluted the relative abundance. However, the relative abundance of *Aspergillus flavus* in PS samples from Shandong province was 9.76%. It suggested that the AF contamination was more related to the relative abundance of *Aspergillus flavus* than other *Aspergillus* species. In addition, the average relative abundances of *Xerochrysium* and *Xeromyces* were higher in samples of Shandong province than those in samples of Anhui and Hebei province. Perhaps the distributions of these two genera might relate to the local storage conditions in Shandong province (34⋅39°N, 114⋅45°E, China), which may be more suitable for the growth of species like *Xerochrysium* and *Xeromyces* than the other sample locations.

As the second most abundant species in the eukaryotic kingdom, fungi include an estimated 2.2 to 3.8 million species (Hawksworth and Lücking, 2017). HTS adds a new prospect to the study of microorganism diversity in many aspects. Compared with traditional identification methods, HTS exhibits its unique advantages. The majority of microorganisms with thousands of sequences can be identified swiftly. Numerous samples can be sequenced simultaneously, considerably saving time. Furthermore, fungal microbiome changes during storage can be monitored (Ercolini, 2013). On the other hand, HTS also features limitations. For example, OTU identity is based on the most abundant sequences, causing the merging of OTUs. This process decreases species identification efficacy (Cunning et al., 2017). Primer bias also results in inability to infer OTU, causing low efficiency in identification (Tedersoo et al., 2015). In addition, although a 97% similarity cut-off is reasonable for known species, no single threshold value for all known and unknown fungal species was simultaneously established within the UNITE database (Blaalid et al., 2013). In the meanwhile, HTS overlooked the phenotype of individual microbes. For example, HTS cannot tell you which *Aspergillus flavus* OTUs are non-aflatoxigenic. Another thing worth mentioning is the selection of genetic marker, and it also has an effect on the result of HTS (Nilsson et al., 2019). In this study, ITS2 exhibited good identification ability in many genera, but it showed relatively poor identification ability for fungal species in *Aspergillus*. *Aspergillus* is a diverse genus with 340 officially accepted filamentous fungal species (Park et al., 2017). As a result of intraspecific diversity, it is difficult to identify the species in *Aspergillus*. Samson et al. (2014) indicated the ITS region was also insufficient for the correct identification of species in *Aspergillus*. Although *MCM7* and *Tsrl* showed good capability to identify species in various fungi, they showed little information about species in *Aspergillus* in NCBI database (Schmitt et al., 2009; Ashtiani et al., 2017). Hence, multiple markers should be applied to identify species in *Aspergillus* in further studies. Although HTS presents certain limitations, it overcomes many deficiencies of traditional methods, and achieves rapid detection of microorganisms. HTS presents a better application prospect in microorganism identification with the further development of this technology.

## Conclusion

The quality and safety of herbs have a significant effect on clinical medication. Cases of fungal contamination and excessive mycotoxin levels in herbs have been consistently reported, preventing their global exportation. Based on previous studies, seed herbs are the most susceptible to mycotoxin contamination. This study highlights the importance of assessing possible AF contamination in common medicinal herbs and the usefulness of HTS to identity any associated AF producing fungi. It serves as a basis for continued scrutiny of ingested herbs to protect consumer health.

## Data Availability Statement

The datasets presented in this study can be found in online repositories. The names of the repository/repositories and accession number(s) can be found in the article/ Supplementary Material.

## Author Contributions

XP: conceptualization, funding acquisition, validation, and writing—review and editing. JY and MG: data curation. JY, MG, WJ, and XP: formal analysis. JY, MG, and XP: methodology. MY: resources. JY and XP: writing—original draft. All authors contributed to the article and approved the submitted version.

## Conflict of Interest

The authors declare that the research was conducted in the absence of any commercial or financial relationships that could be construed as a potential conflict of interest.

## Footnotes

1 http://drive5.com/uparse/

2 http://www.Drive5.Com/Usearch/

## Supplementary Material

Supplementary Figure 1 HPLC chromatograms of **(A)** mixed standard solution of B + G aflatoxins, and **(B)** a solution for PS sample BZ2.

Supplementary Figure 2 LC-MS/MS chromatograms of **(A)** a mixed standard solution of B aflatoxin, and **(B)** a solution for PS sample BZ2.

Supplementary Table 1 Distribution of OTUs for 11 PS samples Y indicates the presence of OTU in this sample; N indicates the absence of OTU in this sample.

Supplementary Table 2 Statistics of α-diversity indices for 11 PS samples.
