# Supplementary figures and images for: Assessment of the Microbiome and Potential Aflatoxin Associated With the Medicinal Herb Platycladus orientalis
Source: Front Microbiol. 2020 Oct 23;11:582679. doi: 10.3389/fmicb.2020.582679 (PMC7644961; doi:10.3389/fmicb.2020.582679)

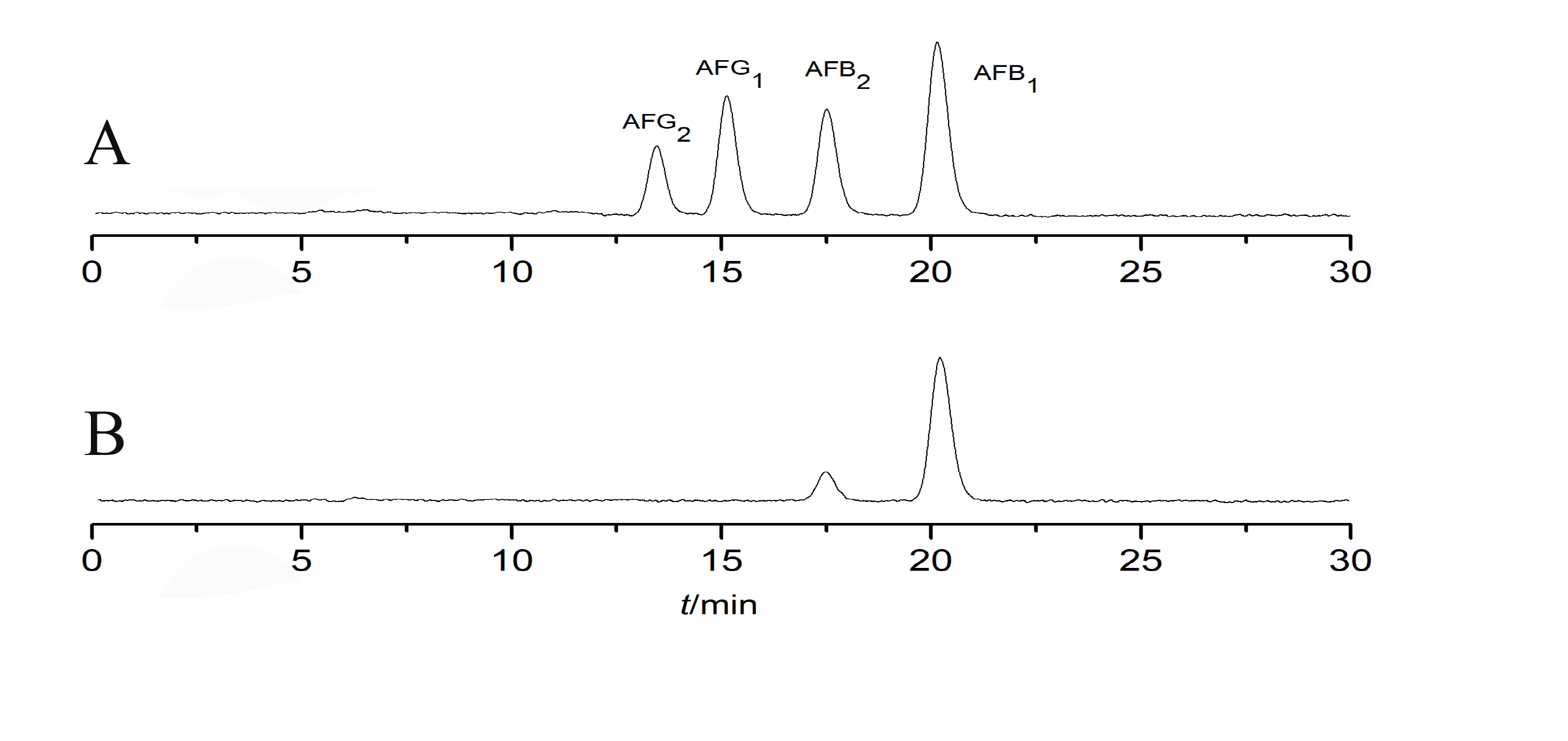

Supplement: Supplementary Figure 1 — HPLC chromatograms of (A) mixed standard solution of B + G aflatoxins, and (B) a solution for PS sample BZ2. [file Image_1.TIF]
