# Supplementary material for: Assessment of the Microbiome and Potential Aflatoxin Associated With the Medicinal Herb Platycladus orientalis
Source: Front Microbiol. 2020 Oct 23;11:582679. doi: 10.3389/fmicb.2020.582679 (PMC7644961; doi:10.3389/fmicb.2020.582679)
Supplement: Supplementary Table 2 — Statistics of α-diversity indices for 11 PS samples. [file Table_2.DOCX]

**Table S2 Statistics of α-diversity indices for 11 PS samples**

| Voucher No. | Chao1 | Goods coverage | Shannon |
| --- | --- | --- | --- |
| BZ1 | 485.90 | 0.9980 | 4.42 |
| BZ2 | 697.09 | 0.9972 | 5.41 |
| BZ3 | 501.80 | 0.9975 | 4.11 |
| BZ4 | 675.18 | 0.9974 | 5.08 |
| BZ5 | 693.89 | 0.9975 | 5.68 |
| BZ6 | 476.13 | 0.9974 | 2.78 |
| BZ7 | 720.87 | 0.9974 | 6.09 |
| BZ8 | 521.93 | 0.9978 | 4.50 |
| BZ9 | 690.36 | 0.9971 | 5.03 |
| BZ10 | 477.88 | 0.9975 | 3.99 |
| BZ11 | 625.00 | 0.9974 | 4.37 |
